# Comprehensive analysis of bioinformatics and system biology reveals the association between Girdin and hepatocellular carcinoma

**DOI:** 10.1371/journal.pone.0315534

**Published:** 2024-12-13

**Authors:** Tengda Huang, Hongying Chen, Hongyuan Pan, Tian Wu, Xiangyi Ren, Liwen Qin, Kefei Yuan, Fang He

**Affiliations:** 1 Division of Liver Surgery, Department of General Surgery and Laboratory of Liver Surgery, and State Key Laboratory of Biotherapy, West China Hospital, Sichuan University, Chengdu, China; 2 Cytology and Molecular Platform, Core Facilities of West China Hospital, Chengdu, China; 3 NHC Key Laboratory of Transplant Engineering and Immunology, Regenerative Medicine Research Center, Frontiers Science Center for Disease-related Molecular Network, West China Hospital of Sichuan University, Chengdu, China; 4 Center of Infectious Diseases, West China Hospital, Sichuan University, Chengdu, China; BMSCE: BMS College of Engineering, INDIA

## Abstract

**Introduction:**

Hepatocellular carcinoma is one of the leading causes of cancer-related mortality worldwide. The actin-binding protein Girdin is overexpressed in various tumors, promoting tumorigenesis and progression. However, the exact mechanisms by which Girdin regulates liver cancer remain poorly understood.

**Methods:**

This study comprehensively analyzed the expression level of Girdin in liver cancer and adjacent tissue, along with the correlation between Girdin expression and the clinical characteristics and prognosis of liver cancer. The analysis integrated data from The Cancer Genome Atlas (TCGA), Gene Expression Omnibus (GEO), and Clinical Proteomic Tumor Analysis Consortium (CPTAC) database. Subsequently, Girdin expression was knocked down to elucidate its role in the progression of liver cancer. Transcriptome sequencing was employed to investigate the mechanistic underpinnings of Girdin’s regulatory impact on liver cancer. Additionally, the Comparative Toxicogenomics Database (CTD) was utilized to identify potential drugs or molecules for liver cancer treatment.

**Results:**

The findings revealed elevated Girdin expression in liver cancer tissues, and heightened Girdin expression correlating with adverse clinical features and prognosis. Silencing of Girdin markedly impeded the proliferation and migration of hepatocellular carcinoma cells. Moreover, transcriptome sequencing demonstrated that silencing Girdin led to differential expression of 176 genes and inhibition of the PI3K/Akt signaling pathway, as well as its upstream pathways—Cytokine-cytokine receptor interaction and Chemokine signaling pathway. Ultimately, we propose that Imatinib Mesylate, Orantinib, Resveratrol, Sorafenib, and Curcumin may interact with Girdin, potentially contributing to the treatment of liver cancer.

**Conclusion:**

This study reveals the association between Girdin and hepatocellular carcinoma, providing novel clues for future research and treatment of hepatocellular carcinoma.

## 1. Introduction

Liver cancer ranks as the third leading cause of cancer-related mortality worldwide, with hepatocellular carcinoma (HCC) being the most prevalent form of primary liver cancer. HCC is characterized by its aggressive behavior, rapid tumor progression, significant challenges in management, limited therapeutic efficacy, and a rising incidence rate [1, 2]. In recent years, substantial progress has been made in the clinical management of liver cancer. Effective treatment modalities currently include surgical resection, radiofrequency ablation, liver transplantation, interventional therapy, radiotherapy, and pharmacotherapy. However, despite these advancements, the five-year survival rate for patients with liver cancer remains a mere 14.1 percent [3], underscoring the ongoing challenges in improving patient outcomes. Therefore, it is imperative to investigate effective biomarkers and understand the mechanisms underlying HCC development.

Girdin, an actin-binding protein often referred to as the "girder" of actin filaments, consists of 1,870 amino acids and has a calculated molecular mass of 220 kilodaltons (kDa) [4]. Girdin modulates intracellular signaling pathways by interacting with key signaling molecules, including the serine/threonine kinase Akt [5, 6], Gαs [7], pyruvate kinase M2 (PKM2) [8], and c-MYC [9]. Alterations in these intracellular signaling pathways can influence cancer cell proliferation, apoptosis, migration, and gene expression, highlighting Girdin’s critical role in tumor development. Furthermore, Girdin serves as a molecular marker for various malignancies and exhibits close associations with diverse prognostic outcomes. Currently, numerous reports have investigated Girdin’s regulatory role in various cancers, including liver cancer [10], non-small cell lung cancer [11], breast cancer [12], colorectal cancer [13], and esophageal cancer [14]. However, the precise mechanism underlying Girdin’s regulation of hepatic carcinoma is yet to be elucidated.

The present study investigated the expression pattern, clinical features, and prognostic significance of Girdin in HCC. Furthermore, the study delved into the impact of transfecting Girdin-specific siRNA on the proliferation and metastasis of the HCC cell line HepG2. This study explored the expression pattern, clinical features and prognostic value of Girdin in HCC. Transcriptome sequencing was used to explore the transcription profiling, differentially expressed genes and pathways involved in Girdin knockdown, so as to understand the function and significance of Girdin in HCC. Overall, this study offers novel perspectives and insights that could contribute to advancements in the diagnosis and treatment of HCC.

## 2. Materials and methods

This study is designed as an experimental research investigation aimed at elucidating the role of Girdin in hepatocellular carcinoma. The methods employed encompass data collection, gene expression analysis, functional validation through gene silencing, and transcriptomic profiling to understand Girdin’s regulatory mechanisms in liver cancer. The location of this study was primarily the research laboratories at West China Hospital.

### 2.1 Data collection and differential analysis

To assess the expression level of Girdin, transcriptome expression matrices of liver hepatocellular carcinoma (LIHC) and normal liver tissue were obtained from The Cancer Genome Atlas (TCGA) database. Subsequently, the dataset GSE144269 [15] was acquired from the Gene Expression Omnibus (GEO) database, and the disparities in Girdin expression between tumors and normal tissues were scrutinized through a paired t-test. The Clinical Proteomic Tumor Analysis Consortium (CPTAC) integrates genomic and proteomic data to identify and characterize all proteins in tumor and normal tissues, thereby helping to discover candidate proteins that can be used as tumor biomarkers.

### 2.2 Clinical characteristics and prognostic value of Girdin

The university of alabama at birmingham cancer data analysis portal (UALCAN) [16] (http://ualcan.path.uab.edu/index.html) is a comprehensive interactive network resource for analyzing cancer Omics data. It enables researchers to collect valuable information and data on the genes that they are interested in. In this study, the clinical characteristics and prognostic value of Girdin were analyzed by using UALCAN.

### 2.3 Cell lines and culture conditions

The human HCC cell line HepG2 was purchased from the Shanghai Cell Bank of the Chinese Academy of Sciences. HepG2 cells were cultured in Dulbecco’s modified Eagle’s medium (HyClone, Logan, UT, USA) containing 10% fetal bovine serum (HyClone), 1% penicillin/streptomycin at an incubator temperature of 37°C and CO_2_ concentration of 5%.

### 2.4 Cell transfection

HepG2 cells were divided into 2 groups, including the negative control group (si-NC) and the si-Girdin group. Based on the human Girdin gene sequence and small interfering RNA (siRNA) sequence with high transfection rate provided by GenBank, Huzhou Hippo Biotechnology Co., Ltd. was commissioned to design the siRNA sequence (5’-3’, GCAAGCUAAGCAAGAUUGAAUTT) and negative control sequence for Girdin. The transient transfection of siRNA was performed using the GenMute™ siRNA Transfection Reagent (SignaGen Laboratories, Maryland, USA) according to the manufacturer’s instructions.

### 2.5 RNA extraction and real-time quantitative PCR (RT-qPCR)

Total RNA was extracted from cells using TRIzol reagent (Invitrogen, USA), RNA purity and concentration were determined, and the A260/A280 ratio of RNA was detected using absorption spectroscopy at 260 nm and 280 nm. The guaranteed ratio is between 1.8–2.1 and the concentration is between 50–500 ng/μL. cDNA was prepared using HiScript III All-in-one RT SuperMix Perfect for qPCR (Vazyme, Nanjing, China). Real-time qPCR was performed using ChamQ SYBR Color qPCR Master Mix (Vazyme, Nanjing, China) according to the manufacturer’s instructions. A method using 2^-ΔΔCt^ with β-action as an internal control was employed. The forward and reverse primers were as follows: Girdin (TCATTGGGAGCAGTTTCGCT and TCCCAAGAAGTGGCTTCGAC); β-actin (AATCTGGCACCACACCTTCT and GTGCCCATCTACGAGGGGTA).

### 2.6 Western blot

The sample was added to the SDS-PAGE gel tank. Depending on the sample size and the number of proteins to be separated, different gel percentages were selected. Electrophoresis buffers were generally Tris-buffers. The voltage was set to 120 V, and the current was 30 mA for 1–2 hours. The gel was removed and transferred to the transfer device. The transferred membrane was placed in a buffer containing the blocker to ensure the membrane surface was adequately covered. The primary antibody (Girdin primary antibody 1:1000, internal reference 1:5000) was incubated with the membrane overnight at 4°C. The membrane was washed with TBST. The membrane was then incubated with the secondary antibody (1:10000) in a buffer containing the cleaning agent for 1–2 hours at room temperature. The membrane was washed with a buffer to remove unbound secondary antibodies, and a chromogenic agent (e.g., ECL) was used to detect the presence of proteins on the membrane.

### 2.7 Cell proliferation

The OLYMPUS CM20 cell culture monitoring system was used to detect the proliferation capacity of HepG2 cells. Fifty thousand cells in suspension with a serum-free suspension were counted and added to a 12-well plate, while 1 mL of Dulbecco’s modified Eagle’s medium (HyClone, Logan, UT, USA) containing 10% fetal bovine serum (HyClone) and 1% penicillin/streptomycin was transferred to a 12-well plate. Continuous incubation occurred for 5 days, and data were collected for statistical analysis.

EdU was used to detect the proliferative capacity of HepG2 cells. Cells were suspended with 500 μl medium, seeded into the 24-well plate (4× 10^4^ cells/well), and cultured until the cells reached the proper growth status. After discarding the original culture medium, 500 μl medium containing 5-Ethynyl-2′-deoxyuridine (EdU) (50 μM) (RiboBio Biotechnology, Guangzhou, China) was added to each well and incubated in cell incubator for 2 hours. The EdU medium was discarded, and the cells were rinsed with PBS twice (5 min each). The cells were then fixed with 4% paraformaldehyde in PBS for 30 minutes at room temperature, quenched in glycine solution (2 mg/mL), permeabilized with 0.5% Triton X-100 for 10 minutes, and subsequently stained with Apollo dye solution and 1X Hoechst 33342 reaction liquid for 30 minutes. Images were captured using the OBSERVER D1/AX10 cam HRC microscope (Zeiss, Oberkochen, Germany) with a charge-coupled device (CCD) camera.

### 2.8 Cell migration and matrigel invasion assays

Transwell membranes (8 μm pore size, 6.5 mm diameter) (Corning Costar, Corning, NY, USA) were used for cell migration and matrigel invasion assays. For transwell migration assay, 4× 10^4^ cells were plated in the top chambers. Serum-free medium was added to the top chambers, while migration-inducing medium (with 10% FBS) was added to the bottom chambers. After 24 hours, the filters were fixed with 4% paraformaldehyde. Cotton swabs were used to scrape the cells on the upper side of the membrane and crystal violet was used to stain the cells on the bottom side of the membrane. The membranes were washed with PBS and photographed after dry out. For matrigel invasion assay, the top chambers were coated with matrigel before 6× 10^4^ cells were plated. Images were taken after 24 hours.

### 2.9 RNA extraction, library construction and sequencing

Sample RNA was extracted using TRIzol reagent (Invitrogen, Carlsbad, CA, USA) according to the manufacturer’s protocol, obtaining 1.5 μg of RNA separately for each sample. The purity and concentration of RNA were assessed on an Agilent 2100 Bioanalyzer (Agilent Technologies, Palo Alto, CA, USA) and checked using RNase-free agarose gel electrophoresis. Once the total RNA sample met the standard, it is enriched with Oligo (dT) beads. Then the purified mRNA was fragmented into short fragments using fragmentation buffer. The first strand of cDNA was synthesized in the M-MuLV reverse transcriptase system using fragmented mRNA as a template and random oligonucleotides as primers. Buffer, dNTPs, RNase H, and DNA Polymerase I were added to synthesize the second strand of cDNA. The purified double-stranded cDNA fragments were end repaired, A bases were added, and ligated to Illumina sequencing adapters. The ligation reaction was purified with the AMPure XP Beads (1.0X). The ligation reaction was purified with AMPure XP Beads (1.0X). Ligated fragments were subjected to size selection by agarose gel electrophoresis and polymerase chain reaction (PCR) amplification. The resulting cDNA library was sequenced using Illumina Novaseq6000 by Gene Denovo Biotechnology Co. (Guangzhou, China).

### 2.10 Bioinformatics analysis

Illumina high-throughput sequencing results were converted to Raw Reads and stored in the FASTQ file format. Low-quality sequences and linker contamination from Raw Reads were removed by the fastp (version 0.18.0) [17] platform for Clean Reads analysis. The short reads alignment tool Bowtie2 (version 2.2.8) [18] was used for mapping reads to ribosome RNA (rRNA) database, and rRNA mapped reads were removed. The filtered-sequencing sequence of each sample was compared to a reference genome (GRCh38) and localized to the genome by the improved BWT algorithm of HISAT2 2.4 [19] software with “-rna-strandness RF” and other parameters set as a default. The number and proportion of genes compared to the genome were called mapping reads and mapping rate. The fragment per kilobase of transcript per million mapped reads (FPKM) value was calculated to quantify its expression abundance, using RSEM software [20]. The differentially expressed genes (DEGs) were identified by DESeq2 software [21] (|log_2_ Fold Change|>log_2_ (1.5) and p-value < 0.05). Pearson correlation analysis, principal component analysis, and gene set enrichment analysis (GSEA) were performed using the OmicShare tool (https://www.omicshare.com/tools/home/report/koenrich.html).

### 2.11 Identification of candidate drugs

The identification of potential drug molecules is one of the most crucial parts of this research. The identification of potential drug molecules was conducted using the Comparative Toxicogenomics Database (CTD, http://ctdbase.org/). CTD is a digital resource that facilitates the study of novel connections in the molecular mechanisms by which chemicals affect health outcomes [22].

### 2.12 Statistical analysis

The primary variables evaluated in this study included Girdin expression levels, clinical characteristics, as well as cellular behaviors like proliferation, migration, and invasion. All data were recorded as mean ± standard error of measurement (SEM). Statistical comparisons were performed using GraphPad Prism statistical software, version 9.5.0. The two-tailed t-test (for two groups) and one-way ANOVA (for multiple groups) were used to identify the significance of the difference. A p-value of less than 0.05 was considered statistically significant. Each experiment was conducted in triplicate to ensure the reliability of the results. Additionally, each experiment was performed by a team of two scientists to maintain consistency in methodology.

## 3. Results

### 3.1 Differential expression of Girdin in hepatocellular carcinoma tissues and normal tissues

**Fig 1:**
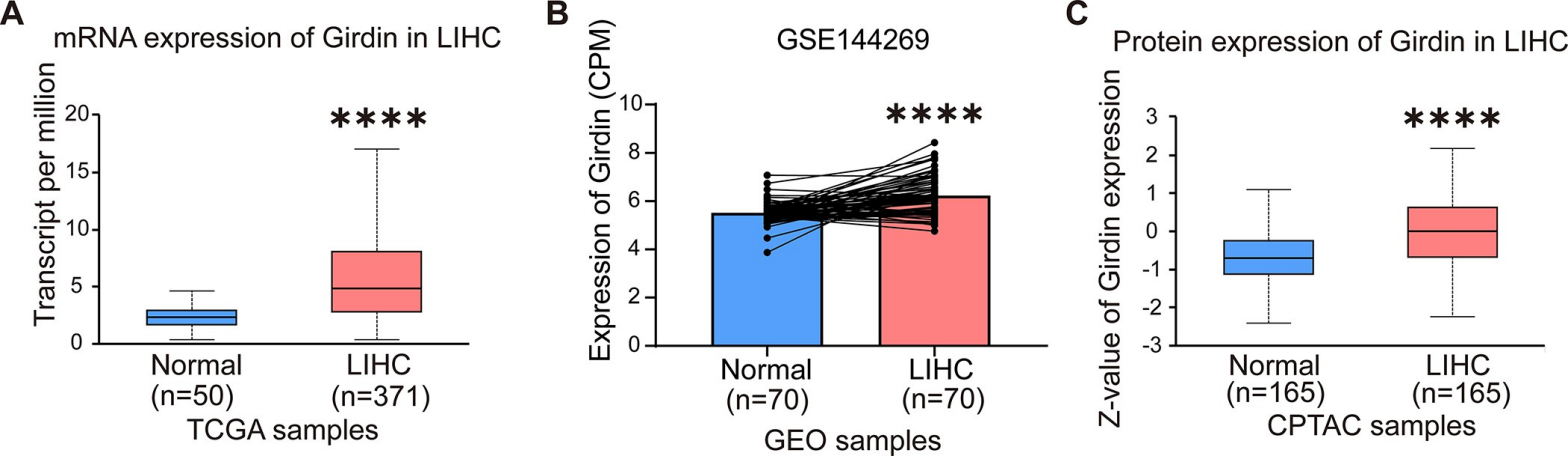
Different mRNA and protein expressions of Girdin in LIHC and normal tissues. (A) Girdin mRNA expression levels in LIHC and normal tissues from the TCGA database. (B) Differential Girdin expression levels in LIHC and normal tissues from the GSE144269 dataset. (C) Girdin protein expression levels in LIHC and normal tissues in the CPTAC database. ****p < 0.0001.

The mRNA expression of Girdin was compared between hepatocellular carcinoma (LIHC) tissues and normal tissues using the TCGA database. Data comprising 371 LIHC samples and 50 normal samples revealed that Girdin mRNA expression was significantly elevated in LIHC compared to normal tissues (Fig 1A). Additionally, the GSE144269 dataset was utilized for validation, showing that Girdin expression was also significantly upregulated in 70 LIHC samples relative to paired adjacent tissues (Fig 1B). Besides, the total Girdin protein expression in LIHC was significantly higher than that in normal tissues from the CPTAC database (Fig 1C). These results indicate that Girdin is differentially expressed in hepatocellular carcinoma tissues and normal tissues.

### 3.2 Clinical features and prognostic value of Girdin expression in hepatocellular carcinoma

**Fig 2:**
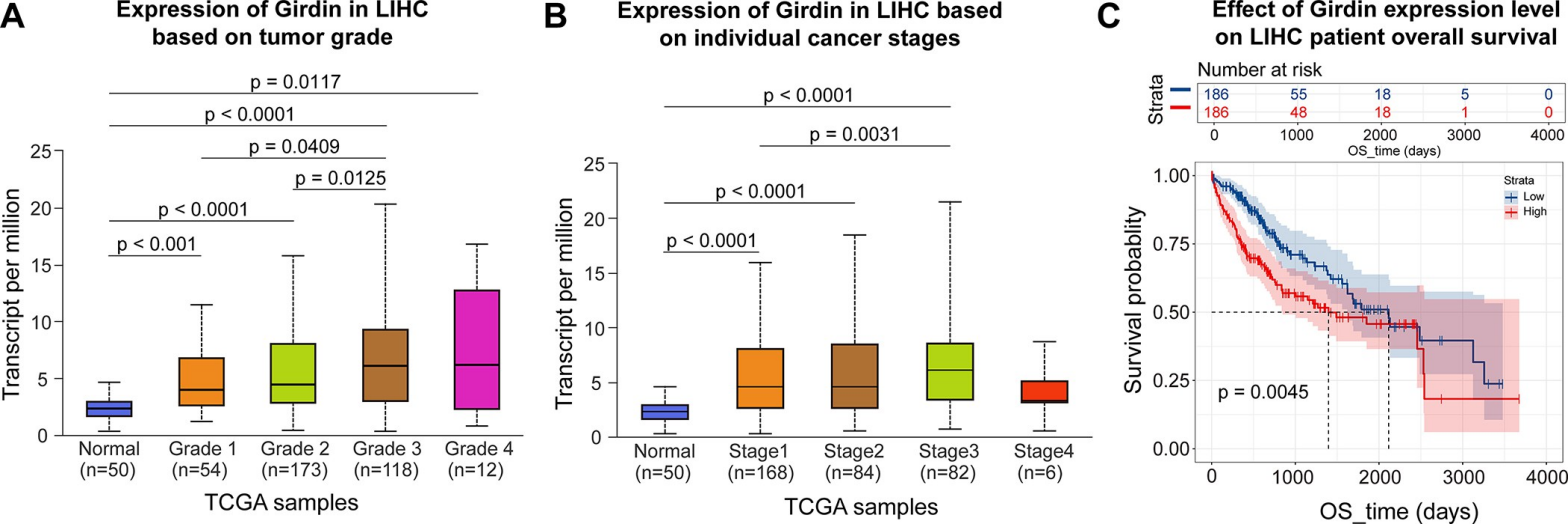
Relationship between Girdin expression and clinicopathological features and prognostic value in LIHC. (A-B) Relative mRNA expressions of Girdin in relation to tumor grade and cancer stage. (C) Kaplan-Meier survival analysis of Girdin expression.

The UALCAN database was employed to assess Girdin expression across different clinical features of LIHC and its correlation with overall survival (OS) in patients. Fig 2A and 2B illustrate that Girdin expression varies with tumor grade and cancer stage. Kaplan-Meier survival analysis further demonstrated that elevated Girdin expression is significantly associated with poor OS in LIHC patients (Fig 2C).

### 3.3 Girdin knockdown suppressed the growth, migration and invasion of hepatocellular carcinoma cells

**Fig 3:**
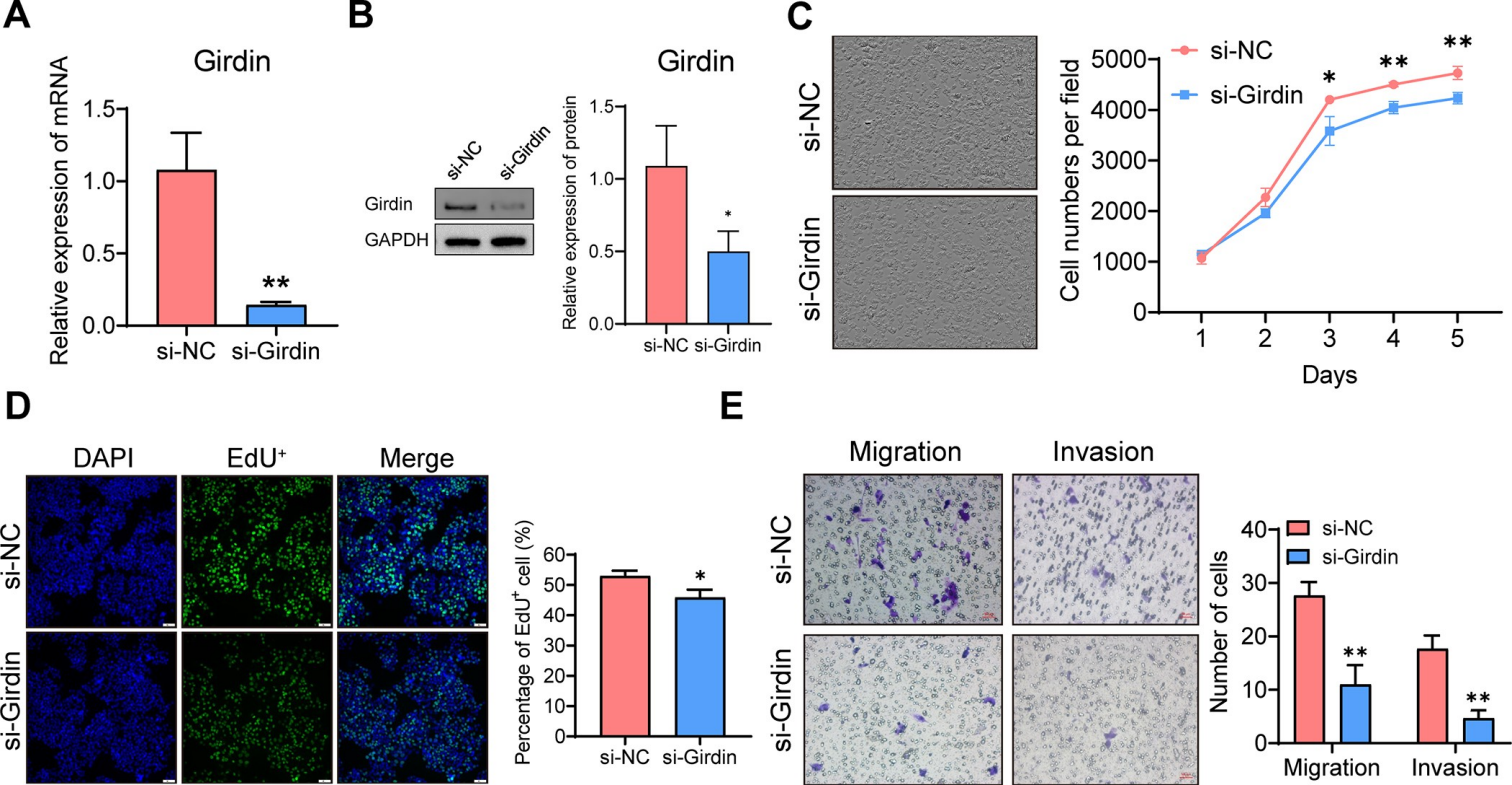
Knockdown of Girdin represses growth and metastasis of hepatoma cells. (A-B) RT-qPCR and western blot of the expression levels of Girdin in HepG2 cells transfected with negative control siRNA or siRNAs targeting Girdin. (C) Cell numbers of HepG2 cells were measured by cell culture monitoring system. (D) DNA replication of indicated HepG2 cells was examined by EdU assay. (E) Cell migration and invasion of HepG2 cells were measured by transwell migration and matrigel invasion assays, respectively. *p<0.05, **p<0.01.

To investigate the regulatory mechanism of Girdin in hepatocellular carcinoma, Girdin-targeting siRNA (si-Girdin) and a negative control (si-NC) were transfected into HepG2 cells. RT-qPCR and Western blot analyses confirmed effective Girdin knockdown (Fig 3A and 3B), enabling subsequent cellular function experiments. Cell proliferation and DNA replication were assessed, revealing that Girdin knockdown significantly inhibited both in HepG2 cells (Fig 3C and 3D). Transwell assays indicated that Girdin silencing markedly reduced migration and invasion compared to the si-NC group (Fig 3E). Collectively, these results demonstrate that Girdin knockdown suppresses malignant behaviors in HCC.

### 3.4 Overview of RNA sequencing data

To explore the potential mechanisms by which Girdin regulates hepatocellular carcinoma, RNA-seq was performed to obtain expression profiles from si-NC and si-Girdin groups. Six cDNA libraries were constructed and analyzed using high-throughput sequencing, with three biological replicates for each group. Six cDNA libraries were constructed and analyzed by high-throughput sequencing, including three biological replicates from the si-NC group (si-NC1, si-NC2, and si-NC3) and three biological replicates from the si-Girdin group (si-Girdin1, si-Girdin2, and si-Girdin3). A total of 235,698,446 raw reads were generated in the six libraries. Specifically, after removing the adapter sequence and low-quality reads, the si-NC group and si-Girdin group produced 36.9–42.3 million and 36.3–41.5 million clean reads, respectively (S1 Table). The percentage of clean reads in all the sample libraries ranged from 99.27% to 99.36%, and approximately 94.26% to 94.76% were uniquely matched to the human reference genome (Homo sapiens, Ensembl_release106) (S1 Table). The average GC content of the six libraries was 48.26%, and the Q20 of each sample was no less than 97.05% (S1 Table), indicating reliable sequencing data for further analysis.

**Fig 4:**
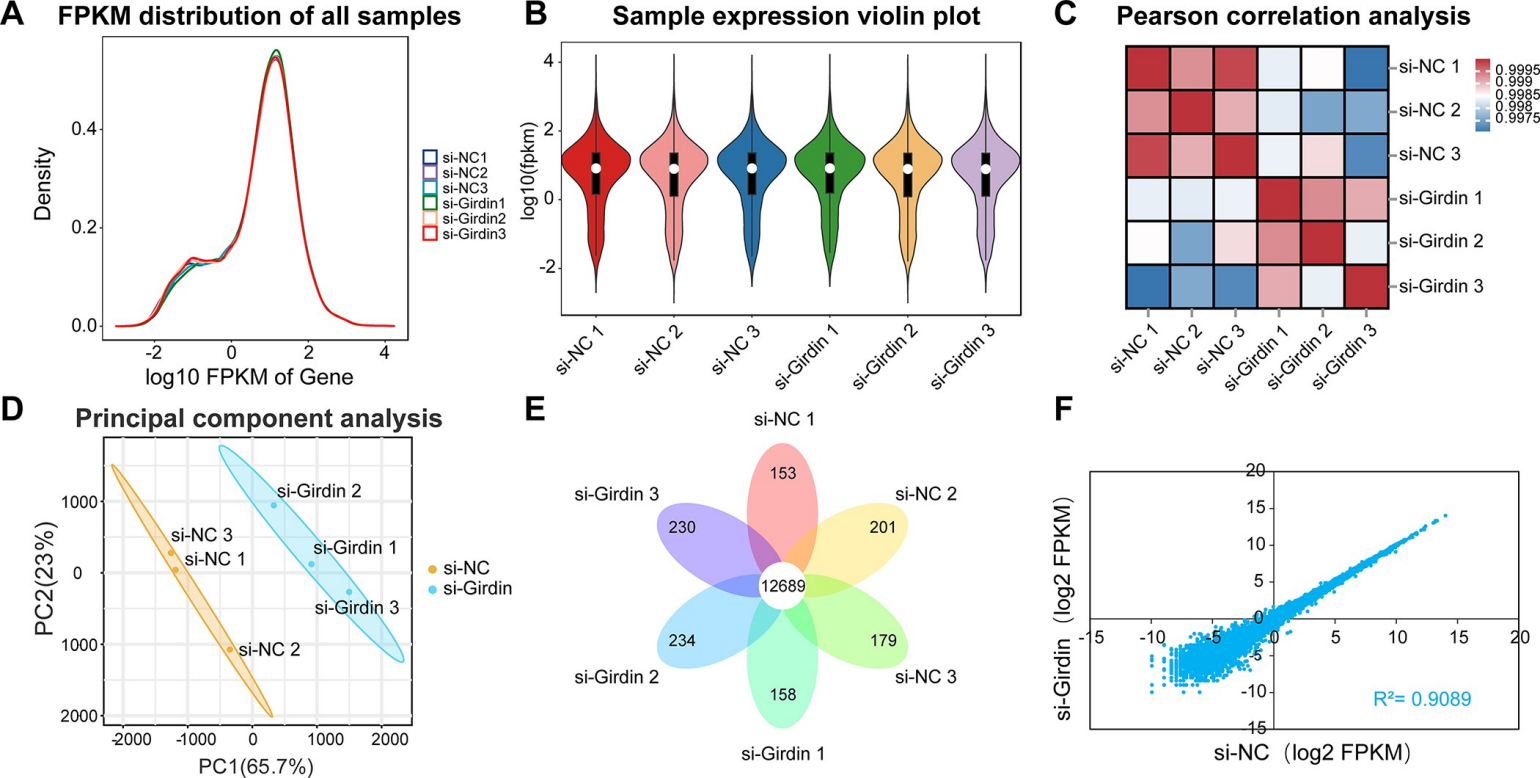
Identification and characterization of mRNA in HepG2 cells. (A) The density distribution of mRNAs was according to log10 FPKM. (B) The violin plot of six sample expressions, which was replaced by log10(FPKM). (C-D) The pearson correlation analysis and principal component analysis of samples. (E) The petal Venn diagram of the gene identified by RNA-seq. (F) Expression abundance correlation analysis between si-NC group and si-Girdin group in RNA-seq.

S2 Table provides the gene expression abundance information of transcriptome. We assessed differences between samples using density profiles of mRNA and violin profiles of expression of 6 samples. The similarity in expression density and expression levels of the six samples indicated their similarity in library construction, sequencing, alignment, and quantification, further supporting the reliability of RNA-seq data (Fig 4A and 4B). The pearson correlation analysis and principal component analysis reveal that the difference of samples between the si-NC and si-Girdin group was obvious, and the similarity of biological replicates was strong (Fig 4C and 4D). Common genes were counted in the si-NC and si-Girdin groups, and 12,689 genes were detected in both groups (Fig 4E). The abundance of genes shared by the si-NC and si-Girdin groups had a high correlation (R^2^ = 0.9089, Fig 4F). The above results show reliable reproducibility of the data in this study.

### 3.5 Identification of differentially expressed genes in hepatocellular carcinoma with Girdin knockdown

**Fig 5:**
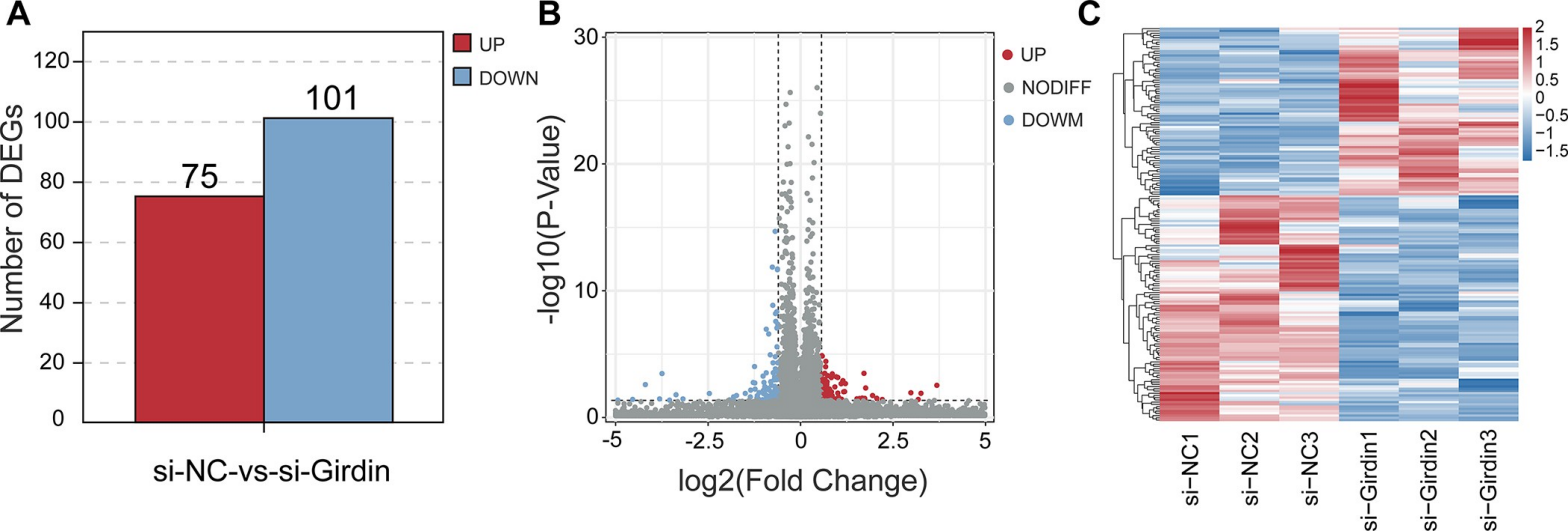
Identification of differentially expressed genes. (A) The statistical analysis of DEGs between si-NC group and si-Girdin group. (B) The volcano map of DEGs. (C) si-NC group and si-Girdin group can be clearly distinguished based on their transcriptome characteristics. The color key (from blue to red) of abundance value indicated low to high expression levels.

Differentially expressed genes were identified based on RNA-seq data, applying thresholds of |log2 Fold Change| > log2(1.5) and p-value < 0.05. A total of 176 DEGs were screened, with 101 genes upregulated and 75 downregulated in the si-Girdin group (Fig 5A and 5B). The information of DEGs is shown in S3 Table. As shown in Fig 5C, DEGs are clustered hierarchically by supervised. Therefore, this study identified significantly differentially expressed genes affected by interference with Girdin expression.

### 3.6 Gene set enrichment analysis revealed that Girdin affected the development and progression of hepatocellular carcinoma by regulating the PI3K-Akt pathway

**Fig 6:**
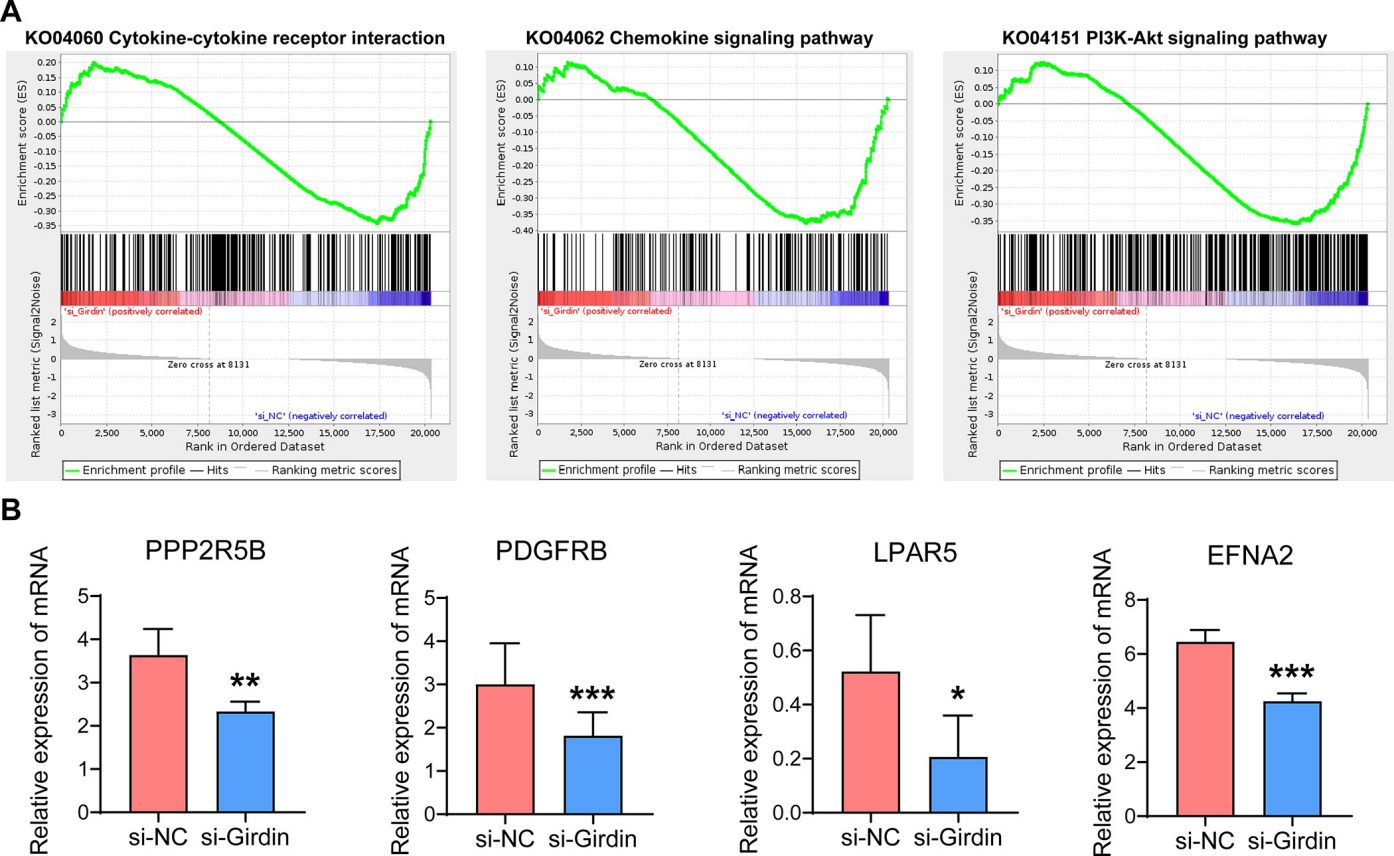
GSEA-KEGG analysis of the transcriptome. (A) The GSEA map of KO04060 Cytokine-cytokine receptor interaction, KO04062 Chemokine signaling pathway, and KO04151 PI3K-Akt signaling pathway. (B) Partial gene expression of PI3K-Akt signaling pathway quantified by RNA-seq. PPP2R5B, protein phosphatase 2 regulatory subunit B’beta. PDGFRB, platelet derived growth factor receptor beta. LPAR5, lysophosphatidic acid receptor 5. EFNA2, ephrin A2. *p<0.05, **p<0.01, ***p<0.001.

To further understand the mechanism by which Girdin regulates hepatocellular carcinoma, gene set enrichment analysis was performed. The results showed that, in the si-Girdin group, several key pathways were inhibited compared to the si-NC group (Fig 6A). Notably, the KO04151 PI3K-Akt signaling pathway was downregulated, along with its upstream pathways—KO04060 Cytokine-cytokine receptor interaction and KO04062 Chemokine signaling pathway—which are known to influence PI3K-Akt signaling. These upstream pathways may explain the broader regulatory effects of Girdin on the PI3K-Akt pathway. In Fig 6B, the downregulation of specific genes within the PI3K-Akt signaling pathway is highlighted, providing further evidence of Girdin’s role in modulating this critical pathway in hepatocellular carcinoma.

### 3.7 Identification of candidate drugs

Protein-drug interaction analysis plays a vital role in medication development [23]. Based on the differentially expressed genes in the PI3K-Akt pathway after silencing Girdin, we used the CTD database to predict five drug candidates, including Imatinib Mesylate, Orantinib, Resveratrol, Sorafenib, and Curcumin. Table 1 depicts the potential drugs in accordance with their interactions. These potential drugs are recommended for the treatment of hepatocellular carcinoma.

**Table 1:** Predicted drugs or chemicals for HCC.

Name	Interactions	Chemical Formula
Imatinib Mesylate	29	C_30_H_35_N_7_O_4_S
Orantinib	9	C_18_H_18_N_2_O_3_
Resveratrol	8	C_14_H_12_O_3_
Sorafenib	7	C_21_H_16_ClF_3_N_4_O_3_
Curcumin	6	C_21_H_20_O_6_

## 4. Discussion

In this study, the expression differences, clinical information and prognostic value of Girdin in LIHC were analyzed. Initially, we employed TCGA, GEO, and CPTAC database analyses, leading to the conclusion that the total mRNA and protein expression of Girdin in LIHC exceeded that in normal tissues. Subsequently, we observed an increase in Girdin expression with advancing staging and grading. The results suggest that elevated Girdin expression promotes tumorigenesis. Furthermore, high expression of Girdin in LIHC is correlated with poor overall survival, predicting an unfavorable prognosis.

Girdin has been reported to have elevated expression in many malignant tumor tissues, such as esophageal, breast, and colon cancers. In this study, we found that knocking down Girdin expression in HCC cells significantly inhibited cell proliferation and metastasis. This is consistent with the results of previous studies [10].

The phosphatidylinositol 3-kinase (PI3K)-protein kinase-B (Akt) signaling pathway is one of the most important intracellular signal transduction pathways, which regulates cell survival, metabolism, proliferation, and angiogenesis [24]. PI3K phosphorylates phosphatidylinositol-4,5-bisphosphate (PIP2) to produce phosphatidylinositol-3,4,5-triphosphate (PIP3), which then recruits oncogenic signaling proteins, including serine and threonine kinase Akt [25]. Once activated, Akt phosphorylates and thus affects many downstream signaling pathways, including mechanistic target of rapamycin (mTOR) [26], Vascular endothelial growth factor (VEGF) [27], mitogen-activated protein kinase (MAPK) [28], NFκB [29], p53 [30], and others. As one of the oncogenic signaling pathways, PI3K/Akt signaling-activated cancers will become more aggressive, and Akt pathway activation has been shown to be an important risk factor for early recurrence and poor prognosis in liver cancer patients [31]. In this study, we found that Girdin silencing in hepatocellular carcinoma cells significantly inhibited the PI3K-Akt signaling pathway, suggesting that the PI3K-Akt signaling pathway mediates the regulatory effect of Girdin on hepatocellular carcinoma progression. In addition, Girdin knockdown significantly inhibited the expression of protein phosphatase 2 regulatory subunit B’beta (PPP2R5B), platelet derived growth factor receptor beta (PDGFRB), lysophosphatidic acid receptor 5 (LPAR5), and ephrin A2 (EFNA2). According to previous reports, PPP2R5B, and EFNA2 can be used as a prognostic biomarker for hepatocellular carcinoma [32–35]. PDGFRB promotes the malignant physiological phenotype of tumors by activating the PI3K/Akt signaling pathway [36, 37]. Besides, LPAR5 regulates cancer cell proliferation, migration, invasion and tumor immunity. For example, knocking down LPAR5 can inhibit thyroid cancer through the PI3K/Akt/mTOR signaling pathway [38]. LPAR5 stimulates the proliferation and migration potential of non-small-cell lung cancer (NSCLC) by positively regulating MLLT11 [39].

Several drugs have been identified as potential treatments for hepatocellular carcinoma based on the differentially expressed genes in the PI3K-Akt pathway following Girdin silencing. Specifically, Imatinib Mesylate, Orantinib, Resveratrol, Sorafenib, and Curcumin have been suggested. Imatinib and Orantinib target multiple protein tyrosine kinases [40, 41]. Resveratrol is a natural polyphenol that has antioxidant, anti-inflammatory, heart-protecting and cancer-fighting properties [42]. Resveratrol induces autophagy and inhibits the progression of human hepatocellular carcinoma by regulating p53 and PI3K/Akt pathways [43]. Sorafenib is the first FDA-approved tyrosine kinase inhibitor (TKI) for advanced hepatocellular carcinoma and is a first-line treatment for HCC patients [44]. Sorafenib can block the activation of MAPK/extracellular-signal-regulated kinase (ERK) signaling and inhibit cancer cell proliferation by inhibiting the expression of subtypes of serine/threonine kinases Raf, RAF-1 and B-Raf [45]. Curcumin has anti-inflammatory, antioxidant and antitumor activities. Studies have shown that curcumin can regulate several pathways, including PI3K/Akt, MAPK, Wnt/β-catenin, JAK/STAT, p53, and NF-κB, to exert inhibitory effects on tumors [46]. While these drugs exhibit various mechanisms of action, further research is necessary to explore their interactions with Girdin and the implications for HCC treatment. The potential synergistic effects of these drugs in combination with Girdin modulation warrant further investigation.

Our study unveils promising avenues for the diagnosis and treatment of liver cancer. Nonetheless, it is not without limitations. The study’s scope is limited to in vitro investigations involving hepatocellular carcinoma cell lines, yielding valuable cellular and molecular insights. Future research should explore the role of Girdin using animal models of liver cancer, including ectopic or in situ tumor models. Additionally, translating experimental results into clinical applications requires substantial further progress.

## 5. Conclusions

This study identified elevated expression of Girdin in liver cancer tissues through comprehensive analyses of TCGA, GEO, and CPTAC databases. The investigation also unveiled a significant correlation between Girdin expression, clinical progression, and prognosis. Physiological phenotypic outcomes demonstrated that the suppression of Girdin through knockdown impeded the proliferation and metastasis of hepatocellular carcinoma. Transcriptome sequencing was employed to elucidate the mechanism underlying Girdin’s regulation of liver cancer progression. The RNA-seq results revealed differential expression of 176 genes and the inhibition of the PI3K/Akt signaling pathway, along with its upstream pathways—Cytokine-cytokine receptor interaction and Chemokine signaling pathway—upon Girdin knockdown. The CTD database was utilized to analyze drugs interacting with differentially expressed genes in the PI3K/Akt signaling pathway. This analysis identified drugs or molecules, including Imatinib Mesylate, Orantinib, Resveratrol, Sorafenib, and Curcumin. These findings will contribute to a deeper understanding of the mechanism by which Girdin influences tumor development in the future.

## Supporting information

S1 Table Summary of RNA-seq data. (XLSX)

S2 Table List of mRNA results from RNA-seq. (XLSX)

S3 Table List of DEGs from RNA-seq. (XLSX)

S1 Raw images The raw blot image data. (PDF)
