# Correction: Comprehensive analysis of bioinformatics and system biology reveals the association between Girdin and hepatocellular carcinoma

**DOI:** 10.1371/journal.pone.0329658

**Published:** 2025-08-01

**Authors:** The PLOS One Staff

There are errors in the author affiliations. The correct affiliations are as follows:

Tengda Huang^1^, Hongying Chen^2^, Hongyuan Pan^1^, Tian Wu^3^, Xiangyi Ren^2^, Liwen Qin^2^, Kefei Yuan^1^, Fang He^4^

**1** Division of Liver Surgery, Department of General Surgery and Laboratory of Liver Surgery, and State Key Laboratory of Biotherapy, West China Hospital, Sichuan University, Chengdu, China, **2** Cytology and Molecular Platform, Core Facilities of West China Hospital, Chengdu, China, **3** NHC Key Laboratory of Transplant Engineering and Immunology, Regenerative Medicine Research Center, Frontiers Science Center for Disease-related Molecular Network, West China Hospital of Sichuan University, Chengdu, China, **4** Center of Infectious Diseases, West China Hospital, Sichuan University, Chengdu, China.

The publisher apologizes for the error.
