# Supplementary figures and images for: Comprehensive analysis of bioinformatics and system biology reveals the association between Girdin and hepatocellular carcinoma
Source: PLoS One. 2024 Dec 13;19(12):e0315534. doi: 10.1371/journal.pone.0315534 (PMC11642971; doi:10.1371/journal.pone.0315534)

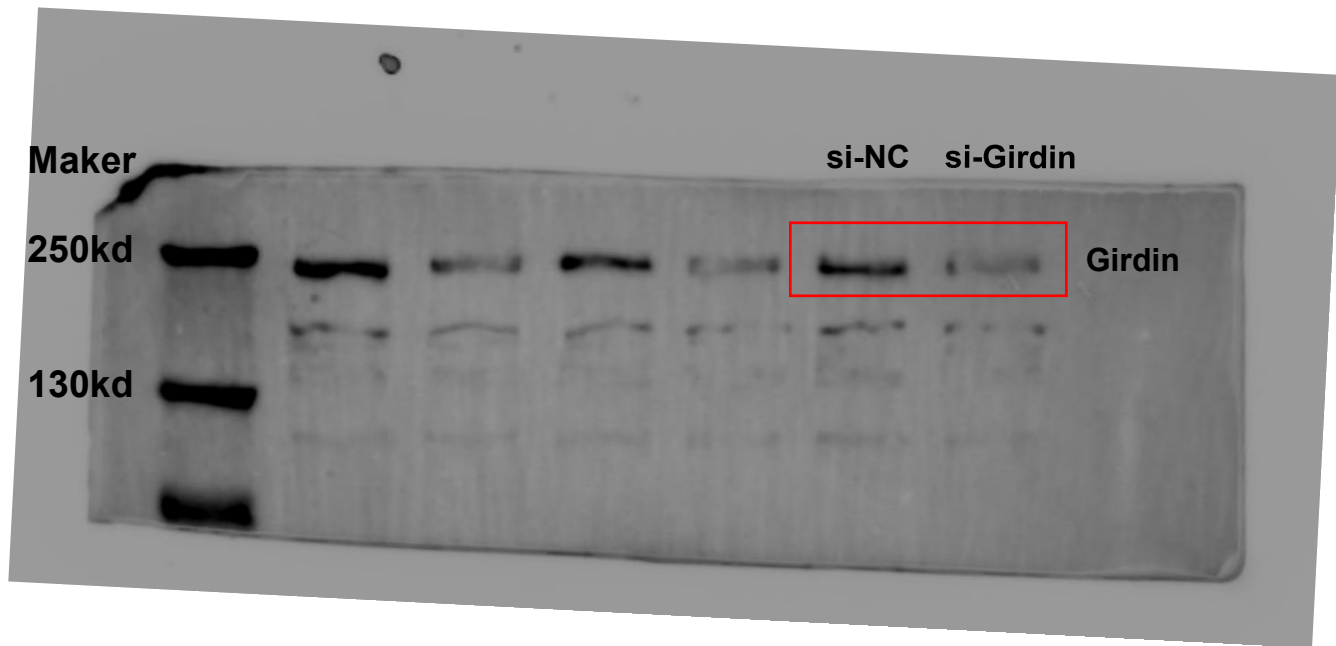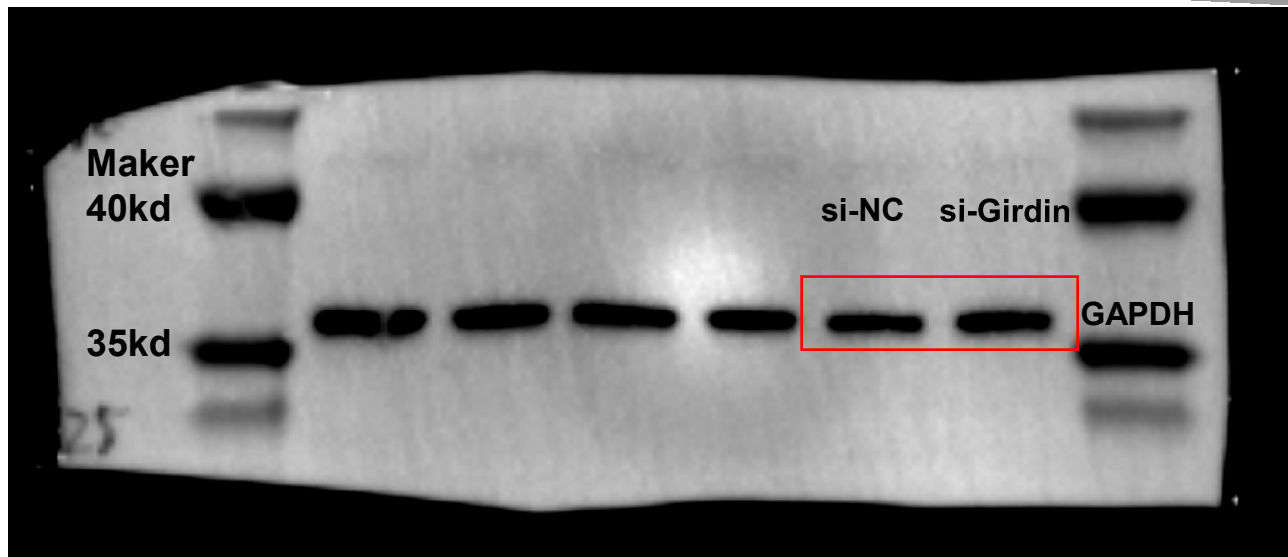

Supplement: S1 Raw images — (PDF) [file pone.0315534.s004.pdf]
